# Die Modelltransferierbarkeit von KI in der digitalen Pathologie: Potenzial und Realität

**DOI:** 10.1007/s00292-024-01299-5

**Published:** 2024-02-19

**Authors:** Robin S. Mayer, Maximilian N. Kinzler, Alexandra K. Stoll, Steffen Gretser, Paul K. Ziegler, Anna Saborowski, Henning Reis, Arndt Vogel, Peter J. Wild, Nadine Flinner

**Affiliations:** 1 Universitätsklinikum, Dr. Senckenbergisches Institut für Pathologie, Goethe-Universität Frankfurt, Theodor-Stern-Kai 7, 60596 Frankfurt am Main, Deutschland; 2 Universitätsklinikum, Medizinische Klinik 1, Goethe-Universität Frankfurt, Frankfurt am Main, Deutschland; 3 Frankfurt Institute for Advanced Studies (FIAS), Frankfurt am Main, Deutschland; 4 Klinik für Gastroenterologie, Hepatologie, Infektiologie und Endokrinologie, Medizinische Hochschule Hannover, Hannover, Deutschland; 5 Wildlab, University Hospital Frankfurt MVZ GmbH, Frankfurt am Main, Deutschland; 6 Frankfurt Cancer Institute (FCI), Frankfurt am Main, Deutschland; 7 University Cancer Center (UCT) Frankfurt-Marburg, Frankfurt am Main, Deutschland

**Keywords:** Künstliche Intelligenz, Cholangiokarzinom, Computerunterstützte Bildinterpretation, Neuronale Netzwerke (Computer), Deep Learning, Artificial intelligence, Cholangiocarcinoma, Computer-assisted image interpretation, Neural networks, computer, Deep learning

## Abstract

**Ziel der Arbeit:**

Künstliche Intelligenz hat das Potenzial, in der Pathologie weitreichende Fortschritte zu erzielen. Doch die tatsächliche Anwendung und Zertifizierung für die Praxis bleibt begrenzt, oft aufgrund von Herausforderungen bei der Transferierbarkeit von Modellen. In diesem Kontext untersuchen wir die Faktoren, die die Transferierbarkeit beeinflussen, und präsentieren Methoden, die dazu beitragen, die Nutzung von KI-Algorithmen in der Pathologie zu verbessern.

**Material und Methoden:**

Mithilfe von Datensätzen aus 2 Instituten und dem öffentlich zugänglichen TCGA-MBIC-Datensatz (TCGA, The Cancer Genome Atlas) wurden Convolutional Neural Networks (CNN) und Vision Transformer (ViT) für Vorhersagen an Urothelgewebe (Karzinom- vs. Normalgewebe) und an intrahepatischen Cholangiokarzinomen (iCCA, „small vs. large duct“) trainiert. Veranschaulicht wird der Einfluss von Farbnormalisierung, Bildartefakten in Training und Anwendung sowie der NoisyEnsemble-Methode.

**Ergebnisse:**

Wir konnten demonstrieren, dass Farbnormalisierung von Gewebeschnitten einen positiven Effekt auf die Interinstitutstransferierbarkeit von CNNs und ViTs hat (respektive +13 % und +10 %) und ViTs meist eine höhere Genauigkeit im externen Test erzielen (hier +1,5 %). Ebenso konnten wir zeigen, dass Artefakte in Testdaten die Vorhersagen von CNNs negativ beeinflusst und dass das Einbeziehen dieser Artefakte während des Trainings zu einer Verbesserung führt. Schließlich erhöhen NoisyEnsembles aus CNNs (besser als aus ViTs) auf verschiedenen Geweben und Fragestellungen die Transferierbarkeit (+7 % Blase, +15 % iCCA).

**Diskussion:**

Wichtig ist vor allem, sich dem Problem der Transferierbarkeit bewusst zu sein: Gute Performance in der Entwicklung bedeutet nicht gute Performance in der Anwendung. Der Einbezug vieler bereits existierender Methoden zur Verbesserung der Transferierbarkeit, wie z. B. Farbnormalisierung und NoisyEnsemble, und deren Weiterentwicklung sind von großer Wichtigkeit.

**Zusatzmaterial online:**

Die Online-Version dieses Beitrags (10.1007/s00292-024-01299-5) enthält eine ausführliche Version der Methoden inkl. Abb. S1 und Abb. S2.

## Abstract (en)

**Objective:**

Artificial intelligence (AI) holds the potential to make significant advancements in pathology. However, its actual implementation and certification for practical use are currently limited, often due to challenges related to model transferability. In this context, we investigate the factors influencing transferability and present methods aimed at enhancing the utilization of AI algorithms in pathology.

**Materials and methods:**

Various convolutional neural networks (CNNs) and vision transformers (ViTs) were trained using datasets from two institutions, along with the publicly available TCGA-MIBC dataset. These networks conducted predictions in urothelial tissue and intrahepatic cholangiocarcinoma (iCCA). The objective was to illustrate the impact of stain normalization, the influence of various artifacts during both training and testing, as well as the effects of the NoisyEnsemble method.

**Results:**

We were able to demonstrate that stain normalization of slides from different institutions has a significant positive effect on the inter-institutional transferability of CNNs and ViTs (respectively +13% and +10%). In addition, ViTs usually achieve a higher accuracy in the external test (here +1.5%). Similarly, we showcased how artifacts in test data can negatively affect CNN predictions and how incorporating these artifacts during training leads to improvements. Lastly, NoisyEnsembles of CNNs (better than ViTs) were shown to enhance transferability across different tissues and research questions (+7% Bladder, +15% iCCA).

**Discussion:**

It is crucial to be aware of the transferability challenge: achieving good performance during development does not necessarily translate to good performance in real-world applications. The inclusion of existing methods to enhance transferability, such as stain normalization and NoisyEnsemble, and their ongoing refinement, is of importance.

Künstliche Intelligenz (KI) hat sich in einer Vielzahl von Anwendungen bewährt, angefangen bei Bildverarbeitung (z. B. Gesichtserkennung) bis hin zu Datenbanken (z. B. zielgerichtete Werbung). Auch in der Pathologie wird an KI geforscht und vor allem im wissenschaftlichen Umfeld konnten bereits Erfolge verbucht werden, die von der Unterstützung einfacher diagnostischer Aufgaben bis hin zur Vorhersage von molekularen Eigenschaften oder dem Überleben von Patienten reichen [1, 2]. Dennoch gibt es in der Pathologie Herausforderungen, die in anderen Bereichen weniger gravierende Folgen haben: Die Transferierbarkeit der Algorithmen auf neue Datensätze.

Unter der Transferierbarkeit versteht man das Anwenden von trainierten KI-Modellen auf Daten, welche nicht Teil des Trainings waren und z. B. aus neuen Instituten stammen. Geprüft wird hierbei, ob die Modelle das Gelernte generalisieren können oder ob sie sich auf spezifische Eigenschaften des Trainingsdatensatzes konzentrieren. Um dies zu testen, ist es gängige Praxis Out-of-domain-Tests zu verwenden, die z. B. Bilder enthalten, die in Instituten erstellt wurden, die noch nicht zum Training beigetragen haben [3]. Im Gegensatz hierzu wird beim Transferlernen die Lösung eines bestimmten Problems auf ein neues Problem übertragen. Das Vortraining von Convolutional Neural Networks (CNN) auf großen Datensätzen ist hier ein prominentes Beispiel [4].

Im Allgemeinen gilt: Je vielfältiger und repräsentativer der Trainingsdatensatz ist, desto wahrscheinlicher ist es, dass das Modell auf Daten mit leicht veränderten Eigenschaften erfolgreich angewendet werden kann und transferierbar ist [5]. Jedoch sind große und vielfältige Trainingsdatensätze aus multiplen Instituten nicht für jede Fragestellung vorhanden, vor allem dann nicht, wenn es um seltene Krankheiten geht [6]. Außerdem sollte bewusst sein, dass nicht immer alle Eventualitäten im Trainingsdatensatz abgebildet sind, da durch neue Entwicklungen immer unvorhergesehene Eigenschaften in den Schnitten auftreten können. Somit ist es wichtig, sich mit der Transferierbarkeit von KI-Modellen auseinanderzusetzen, um zu verstehen, wie diese beeinflusst wird und optimiert werden kann.

## Realität der KI-Integration in der digitalen Pathologie

Die Anzahl der für die Pathologie entwickelten KI-Methoden steigt rasant. In PubMed wurden in 2000 nur 77 Artikel, in 2010 schon 453 und in 2020 bereits 3108 Artikel veröffentlicht, die auf die Suchanfrage „pathology AND (artificial intelligence OR machine learning)“ passen. Diese Zahlen zeigen, dass KI eine wichtige Rolle in der Pathologie einnimmt und dazu beitragen kann, verschiedene Probleme zu lösen:

1. Reduzierung von Kosten, wenn mittels KI bestimmt wird, welche Labortests sinnvoll sind [1, 7],
2. vermindern von Intra- und Interbeobachtervariabilität und das Schaffen neuer Standards, wenn mittels KI z. B. das Schätzen von betroffenen Zellen (z. B. Anteil Ki-67+-Zellen) unterstützt wird [8],
3. Zeitersparnis, wenn die KI z. B. alle Schnitte eines Falls nach Relevanz sortiert oder hoch repetitive Aufgaben, z. B. Auszählen von Objekten, unterstützt [8, 9],
4. vermeiden von Fehlern und Übersehen von seltenen Diagnosen, da niemand Experte für alles ist [6].

Trotzdem gibt es erst einen von der Food and Drug Administration (FDA) zugelassenen KI-Algorithmus in der Pathologie, welcher Inferenz von KI in der Anwendung nutzt ([10], Stand FDA: Okt. 2023). In der Radiologie hingegen gibt es bereits 531 FDA-zugelassene Algorithmen und weniger Transferprobleme, da hier z. B. bestehende Standardisierungssysteme wie DICOM („Digital Imaging and Communications in Medicine“) angewendet werden [11]. Beim Beispiel aus der Pathologie handelt es sich um das KI-Tool Paige Prostate [2], welches die Unterstützung von Ärzten während der Routinediagnostik ermöglicht. Die FDA-Zertifizierung war allerdings ein weiter Weg: Erst nach mehreren Jahren, genauester Analyse von Daten aus 218 verschiedenen Instituten und der Kontrolle durch 16 Pathologen erfolgte die Marktfreigabe durch die FDA [12].

Dies verdeutlicht, wie schwierig es ist, ausreichend genaue und robuste Algorithmen für die Pathologie zu entwickeln, die auch auf Daten aus anderen Instituten anwendbar sind. Da Fehler bei Entscheidungen von KI gerade im medizinischen Bereich gravierende Auswirkungen haben können, muss mit den bekannten Herausforderungen sorgfältig umgegangen werden. Neben Punkten wie der Erklärbarkeit oder der Bewertung der Güte von Vorhersagen hat die Transferierbarkeit der Algorithmen auf neue Datensätze eine besondere Relevanz.

Auch im wissenschaftlichen Umfeld nutzen immer mehr Studien externe Testdaten, um zu prüfen, ob ihre Modelle generalisieren [5].

Jedoch ist es nicht nur wichtig, empirisch zu überprüfen, ob ein Modell generalisiert, sondern auch zu verstehen, welche Parameter die Übertragbarkeit verbessern und wie das Problem überwunden werden kann. Im Folgenden werden, auch anhand von eigenen Beispieldaten, diese Faktoren genauer beleuchtet und Methoden vorgestellt, die den Transfer von Modellen verbessern.

## Einflussfaktoren auf Transferierbarkeit

**Figure 1:**
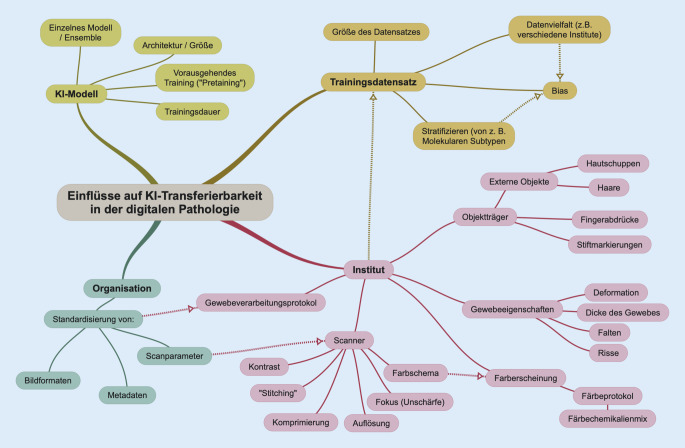

Sollen Daten genutzt werden, welche vom Trainingsdatensatz abweichen, verlieren Modelle häufig Genauigkeit [13]. Dies kann von vielen Faktoren beeinflusst werden (Abb. 1):

1. Ein wichtiger Faktor ist die *Modellarchitektur*, einschließlich ihrer Größe und der Anzahl der Layer. Generell gilt, dass größere Modelle i. d. R. ein größeres Potenzial haben, zu generalisieren. Sie benötigen jedoch auch mehr Daten zum Trainieren [14]. Dabei kann die Datenerweiterung (engl. „data augmentation“) helfen, bei der bestehende Daten durch z. B. Rotation leicht verändert werden [4]. Zusätzlich kann ein Vortraining (engl. „pre-training“) helfen. Hier wird das Modell vor dem eigentlichen Training auf Daten trainiert, die möglicherweise nicht direkt mit der Zielaufgabe zusammenhängen [15].
  Die Dauer des Trainings ist ein weiterer entscheidender Faktor. Eine zu kurze Trainingsdauer kann dazu führen, dass das Modell nicht genügend Features erlernt und nicht generalisiert. Andererseits kann ein zu langes Training dazu führen, dass das Modell die Trainingsdaten auswendig lernt (engl. „overfitting“) und neue Datenpunkte nicht korrekt zuordnen kann [5]. Durch frühzeitiges Anhalten des Trainings (engl. „early stopping“) bei stagnierender Performance kann dieses Problem weitgehend vermieden werden.

2. Der *Trainingsdatensatz* spielt ebenfalls eine entscheidende Rolle bei der erfolgreichen Übertragbarkeit von Modellen. Zum einen ist die Größe des Datensatzes von Bedeutung [16]. In der digitalen Pathologie ist es oft eine Herausforderung, ausreichend große Mengen an annotierten Daten zu erhalten. Teilweise werden zeitaufwendige manuelle Annotationen benötigt [17], es fehlen Metadaten (z. B. Sequenzierungs- und/oder Überlebensdaten) oder das Einverständnis der Patienten. Aber auch Scandauer und permanente Speicherung hochauflösender Bilder digitalisierter Schnittpräparate stellen Hindernisse dar [18]. Zum anderen kann es auch bei einem großen, zu einheitlichen und wenig variablen Trainingsdatensatz passieren, dass Modelle nicht genügend Features erlernen und nicht generalisieren [19].
  Ein ungewollter Bias kann ebenfalls leicht im Trainingsdatensatz entstehen, wenn z. B. Daten nicht aus verschiedenen Einrichtungen kommen oder sogar Daten einzelner Subgruppen bevorzugt aus einer spezialisierten Einrichtung gewählt werden, in denen Patienten nur mit bereits bestehendem Verdacht behandelt werden. Dann können sich Modelle schnell an Mustern orientieren, die nur im Trainingsdatensatz auftreten [19]. Weiterhin kann ein Bias im Datensatz entstehen, wenn die Trainingsdaten bevorzugt aus bestimmten Regionen (z. B. Deutschland) stammen und dadurch nicht alle ethnische Gruppen repräsentiert sind, auf die der Algorithmus möglicherweise angewendet werden soll.
3. Die Übertragung von Modellen kann auch durch *organisatorische* Faktoren beeinflusst werden, wie z. B. die Standardisierung von Bildformaten und Metadaten sowie die Einstellungen der Scannerparameter [5]. Hier könnten auch Standards wie DICOM eine Übertragung vereinfachen. Anders als in der Radiologie gibt es in der Pathologie jedoch nur eine sehr geringe Adaption [11].
4. Schließlich können bei der Verarbeitung von Geweben und Objektträgern im *Institut* verschiedene Effekte und Artefakte auftreten. Dabei spielt z. B. die Wahl des Scanners sowie dessen Konfiguration eine Rolle. Zur Konfiguration gehören Punkte wie Farbschema, Kontrast, Kompressionsstärke, Auflösung, Anzahl der Fokuspunkte und das Stitching (Zusammenfügen kleiner gescannter Bereiche zum Gesamtbild). Zudem können physische Merkmale der Glas-Slides wie das Vorhandensein fremder Objekte auf dem Objektträger (z. B. Haare, Hautschuppen) sowie Veränderungen am Gewebe, die während der Verarbeitung auftreten können, die Übertragbarkeit erschweren. Dazu gehören Deformationen des Gewebes, zu dünne/dicke Schnitte, Falten sowie Risse im Gewebe [20].

## Material und Methoden

In dieser Arbeit haben wir die Übertragbarkeit von trainierten Modellen anhand zweier Beispiele untersucht. Für die Unterscheidung zwischen Karzinom- und Normalgewebe (Urothelgewebe) wurden Whole Slide Images (WSI) aus dem TCGA („The Cancer Genome Atlas“; *n* = 107) für Training, Validierung und den internen Test verwendet. Für jedes WSI wurden 80 Kacheln (299 × 299 Pixel mit einer Auflösung von 1 µm/Pixel) jeder Klasse gesampelt und patientenstratifiziert auf die Datensätze aufgeteilt. Als externer Test wurden Daten (*n* = 17) aus dem Dr. Senckenbergischen Institut für Pathologie (SIP) genutzt, um die Generalisierbarkeit der Modelle zu überprüfen. Im zweiten Bespiel wurde zwischen histologischem „small duct“ und „large duct“ des intrahepatischen Cholangiokarzinoms (iCAA) unterschieden. WSIs aus dem SIP (*n* = 62) wurden für Training, Validierung und internen Test genutzt. Eine weitere Kohorte (*n* = 25) aus der Medizinischen Hochschule Hannover (MHH) diente als externer Test.

Die Modelle wurden für 25 Epochen mit TensorFlow [21] trainiert und umfassten CNNs (ResNet18, DenseNet121, VGG16 und Xception) und Vision Transformer (ViT-B/16). Als Optimierungsalgorithmus wurde AdaMax mit einer Lernrate von 0,001 genutzt, als Verlustfunktion („loss“) wurde die binäre Kreuzentropie („binary cross-entropy“) gewählt. Für Ensembles wurden je 15 Modelle durch Bagging trainiert und aggregiert. Bei NoisyEnsembles wurden zusätzlich 15 % der Labels im Training verfälscht [13].

Die Farbnormalisierung der Kacheln (Training + Test) wurde nach Vahadane [22] durchgeführt, für die Farbaugmentierung (nur Training) wurde die Hue-Saturation-Value-Methode (HSV) [23] genutzt. Die untersuchten Bildartefakte wurden mit dem Python-Modul cv2 (für Unschärfe, Helligkeit und Kontrast) und Pillow (für JPEG-Komprimierung) nach der Farbnormalisierung in das Bild eingebracht.

Für eine ausführlichere Version der Methoden mit allen verwendeten Parametern wird auf den Text im *Onlinezusatzmaterial *verwiesen.

## Ergebnisse und Diskussion

### Farbnormalisierung verbessert Übertragbarkeit von CNNs

**Figure 2:**
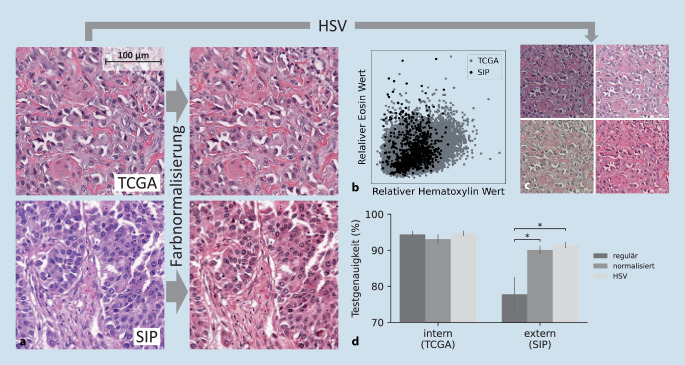

Eine etablierte Methode zur Überwindung von Transferproblemen in der digitalen Pathologie ist die Farbnormalisierung (FN) (engl. „stain normalization“) [24], die Farbunterschiede aus Digitalisierung und Labor ausgleichen kann (Abb. 2a). Bei der Vahadane-FN [22] werden zuerst die Hämatoxylin- und Eosin-Konzentrationen approximiert (Abb. 2b) und an ein Referenzbild angeglichen. Die FN verändert in unserem Beispiel zur Unterscheidung von Karzinom- vs. Normalgewebe im muskelinvasiven Urothelkarzinom die Genauigkeit im internen Test (der genau wie Trainings- und Validierungsdatensatz Daten aus der TCGA-Kohorte [25] enthält) nicht. Die Patienten wurden stratifiziert auf die verschiedenen Datensätze aufgeteilt. Im Transfer auf Daten aus einem anderen Institut (hier dem SIP) kommt es aber zu einer signifikanten Erhöhung der Genauigkeit (+13 %, Abb. 2d). Neben Vahadane gibt es noch weitere FN-Methoden, z. B. werden KI-Modelle wie StainGan [26] für den Farb- und Styletransfer genutzt. Diese funktionieren oft besser, benötigen aber zum Trainingszeitpunkt Beispiele der Zieldomain und können Artefakte in die Bilder einfügen. Die absichtliche Augmentation mit verschiedenen Farbschemata (z. B. HSV) [23] wird nur während des Trainings angewendet. Sie ruft durch Änderungen an Hue und Sättigungswerten Änderungen im Farbschema hervor (Abb. 2c), erzielt genau wie die FN signifikant höhere Genauigkeiten im externen Test (Abb. 2d) und verbessert somit den Transfer der Modelle (+14 %). Dabei scheint HSV einen Vorteil gegenüber der FN zu haben, der jedoch nicht statistisch signifikant ist (Abb. 2d). Aufgrund der ähnlichen Performance können daher beide Methoden genutzt werden.

### Bildartefakte verschlechtern die Transferierbarkeit von CNNs

**Figure 3:**
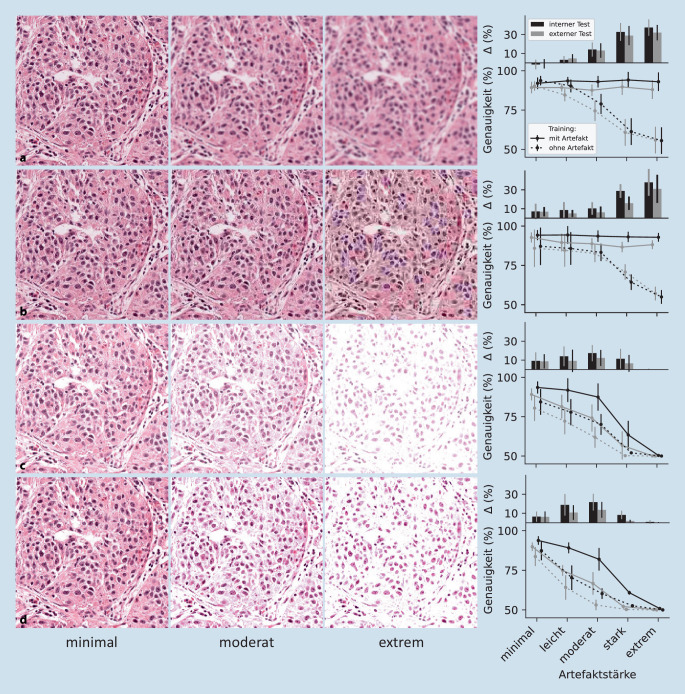

Um zu untersuchen, welchen Einfluss institutsbedingte Eigenschaften auf die Transferierbarkeit haben, wurden verschiedene Artefakte in die Bilder eingefügt:

1. Ein nicht korrekter Fokuspunkt des Scanners wurde durch Gauß-Unschärfe (engl. „blur“) simuliert (Abb. 3a).
2. Um die Dateigröße von WSIs zu minimieren, werden sie als JPEG-komprimierte Bilder gespeichert. Um zu sehen, welchen Einfluss verschiedene Kompressionsraten haben, wurden die Bilder mit verschiedenen Raten komprimiert (Abb. 3b).
3. Verschiedene Helligkeiten (Abb. 3c) und Kontraste (Abb. 3d) können durch Eigenschaften der Slide/des Scanners bedingt sein.

War das Artefakt nur im Test, verschlechterte sich die Performance mit zunehmender Stärke für alle Artefakte auf bis zu 50 % (Abb. 3). War das Artefakt jedoch im Training anwesend, konnte ein Teil der Performance erhalten werden. Bei „blur“ und JPEG-Kompression gab es sogar bei starkem Effekt keinen Qualitätsverlust. Der Performancegewinn (∆ = Genauigkeit_+Artefakt_ – Genauigkeit_-Artefakt_) ist für den internen Test immer höher als für den externen Test. Somit sind Modelle beim Transfer entsprechend anfälliger für Artefakteinflüsse, selbst wenn diese im Training berücksichtigt wurden.

### Methoden zu Verbesserung der Transferierbarkeit von CNN-Modellen

Die Vielseitigkeit der Probleme der Transferierbarkeit haben wir eingehend erläutert, aber wie können diese überwunden werden? Guan et al. [3] beschreiben 2 Methodengruppen, um einen erfolgreichen Transfer oder die auch sog. Domain Adaptation zu erreichen: Shallow Models und Deep Models. Shallow models beruhen auf Statistik und klassischem Machine Learning. Darunter fällt zum Beispiel das Instance Weighting, bei dem die Bilder der Quelldomäne, z. B. anhand ihrer Ähnlichkeit der Featureverteilung zur Zieldomäne, gewichtet werden. Beispiel für Deep Models ist u. a. das Image Alignment. Hier werden Eigenschaften der Bilder (z. B. die Menge des Bildrauschens) durch Deep-Learning-Modelle angeglichen, bevor eine Klassifizierung durchgeführt wird. Die meisten dieser Methoden fokussieren sich jedoch auf die Anpassung an eine bekannte Zieldomäne. Es ist also erforderlich, vor dem Training zu wissen, dass unterschiedliche Domänen existieren und Beispielbilder für beispielsweise das Finetuning oder das Trainieren eines Generative Adversarial Networks (GAN) oder Domänendiskriminators zu haben. Wenn der Domänenshift jedoch unerwartet auftritt, z. B. durch eine veränderte Qualität der Schnitte [13] aufgrund veränderter Arbeitsabläufe im Labor, können diese Methoden zur Domainadaption ohne Neukalibrierung versagen. Daher ist es wichtig auch allgemeine Methoden zur Transferverbesserung anzuwenden, die ohne Wissen über Zieldomänen auskommen, wie z. B. FN oder HSV-Augmentierung. Auch Ensembles, die Kombination mehrerer Modelle zu einer Vorhersage, sind für die Verbesserung der Vorhersagegenauigkeit geeignet, da diese besser auf externe Datensätze übertragen werden können und zum Training keine Informationen über die Testdatensätze benötigen [16, 27].

**Figure 4:**
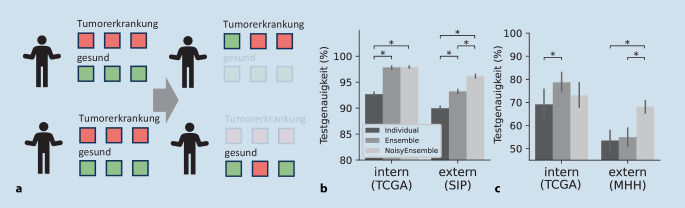

Beispielhaft ist hier die Variation von Ensembles genannt, die das Potenzial hat, die Übertragbarkeit in vielen Situationen zu verbessern. Beim NoisyEnsemble [13] wird für jedes Modell des Ensembles ein neues Subset von Patienten und für jeden Patienten nur je eine Klasse gewählt. Auf dieser Klasse werden die Labels verändert, also ein Noise eingefügt (Abb. 4a). Bei den Urothelkarzinommodellen konnten wir mit dem CNN-NoisyEnsemble den Transfer für den externen Datensatz signifikant um 3 % im Vergleich zum einfachen Ensemble verbessern. Auf dem internen Testdatensatz ist kein Unterschied erkennbar (Abb. 4b). Ebenfalls wurde das CNN-NoisyEnsemble für die Vorhersage von histologischen Small-Duct- und Large-Duct-Typen des intrahepatischen Cholangiokarzinoms (iCCA) getestet. Trainiert wurde hier auf WSI-Daten des SIP, wobei ausreichend WSIs für einen unabhängigen internen Testdatensatz zurückgehalten wurden. Ein externer Datensatz wurde von der Medizinischen Hochschule Hannover zur Verfügung gestellt. Beim internen Test konnte eine Ensemblegenauigkeit von etwa 79 % erreicht werden, extern sinkt diese jedoch auf etwa 53 % (Abb. 4c). Durch Verwendung des NoisyEnsembles konnte die Genauigkeit des externen Tests im Vergleich zum einfachen Ensemble signifikant um ~13 % gesteigert werden, während der interne Test statistisch unverändert blieb. Somit konnte beim iCCA der Transfer auf die neue Domäne ermöglicht werden.

### ViT-Modelle generalisieren oft besser als CNN-Modelle

Unterschiedliche Modelle können zu unterschiedlich guter Transferierbarkeit führen. So auch in unserem Beispiel, in welchem wir mit einer limitierten Datenmenge zum Urothelkarzinom tiefere CNN-Modelle mit höherer Performance im internen Test (Xception > Densenet121 > ResNet18 >> VGG16; Abb. S1) erreichen. Allerdings ist hier auch die Differenz zum externen Test höher als für kleinere Modelle. ResNet18 zeigt eine sehr gute Balance zwischen Performance und Transferierbarkeit (Abb. S1) für die gegebene Fragestellung und Datenmenge.

**Figure 5:**
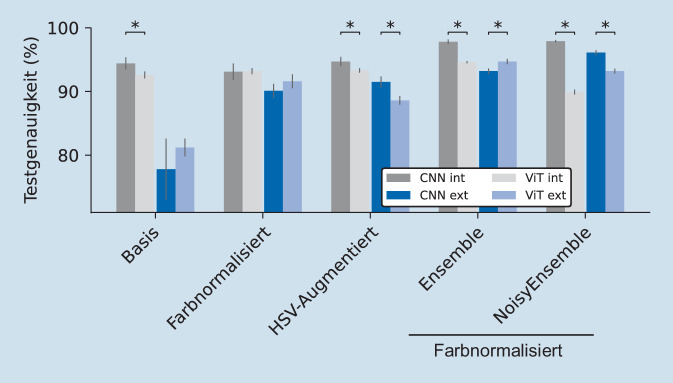

Neben CNNs gibt es in der KI-basierten Bildverarbeitung auch neuere Modellarchitekturen, die zunehmend in den Fokus rücken: Vision Transformer (ViT) [28]. In der Unterscheidung von Karzinom- und Normalgewebe im Urothelgewebe erreichten die individuellen ViTs im internen Test jedoch meistens eine geringere Genauigkeit als CNNs (92 %). Dafür war die Performance der einzelnen Modelle auf dem externen Testdatensatz (81 %) meist höher (Abb. 5), womit ihre Transferierbarkeit besser war. Die ViTs haben also vermutlich eine geringere Anfälligkeit, die Trainingsdaten zu overfitten, und generalisieren besser. Auch ist die FN wichtig, um die Modelle zu verbessern. Beim Training mit FN übertrafen die ViTs signifikant die klassischen CNNs um 2 %im externen Test (~92 %). Die HSV-Augmentierung hingegen verbesserte zwar ebenfalls die Transferierbarkeit (ViT: extern ~88 %), die Performance im externen Test blieb aber trotzdem hinter den CNNs (Abb. 5) und ist somit nicht sinnvoll in Verbindung mit ViTs.

Auch das Bilden von ViT-Ensembles ist ratsam: Die Performance wurde im Vergleich zum individuellen Modell leicht erhöht und interner und externer Test erreichten eine Genauigkeit von ~95 % (Abb. 5). Somit gab es keine Verschlechterung der Performance im Transferfall und ViT-Ensembles stellen eine potenziell robuste Methode für die Transferierbarkeit zwischen verschiedenen Datensätzen dar. Trotzdem erzielten CNN-Ensembles auf dem internen Test die höhere Genauigkeit (97 %, Abb. 5).

Ein NoisyEnsemble aus ViTs ist jedoch nicht sinnvoll. Hier sank die Genauigkeit im Vergleich zu den ViT-Ensembles und die CNN-NoisyEnsembles erreichten insgesamt die höchste Genauigkeit für die Unterscheidung von Karzinom- und Normalgewebe der Blase auf beiden Testdatensätzen (intern 98 %, extern 96 %, Abb. 5). Generell ist bekannt, dass Label Noise bei CNNs vor allem die globalen Features, die in hinteren Layern gelernt werden, beeinflusst [29, 30]. ViTs hingegen lernen globale Features in allen Layern [30] und werden somit ganzheitlich von Label Noise beeinflusst, was das veränderte Verhalten erklären kann. Auch bei der Unterscheidung von histologischem Small-Duct- und Large-Duct-Typen im iCCA zeigt sich das Potenzial von ViTs: Im externen Test war die Genauigkeit der ViT-basierten Vorhersagen entweder höher oder mit CNN-basierten Vorhersagen vergleichbar. Im internen Test ist die Genauigkeit jedoch wieder niedriger (Abb. S2).

Somit sollte die Modellarchitektur mit Bedacht gewählt werden. CNNs erreichen im internen Test meist höhere Genauigkeiten und eignen sich sehr gut für die NoisyEnsemble Methode. Die Transformer wiederrum, erreichen zwar geringere maximale Genauigkeiten im internen Test, aber konnten im Ensemble teilweise ohne Verlust von Genauigkeit auf einen neuen Datensatz transferiert werden. Für ein NoisyEnsemble sind ViTs jedoch weniger geeignet.

## Fazit für die Praxis

- Künstliche Intelligenz wird zukünftig eine große, unterstützende Rolle in der Pathologie einnehmen. Dem im Weg stehen bislang vor allem die Zertifizierung und das damit zusammenhängende Sicherstellen der Transferierbarkeit von Machine-Learning-Algorithmen.
- Die Transferierbarkeit wird von Faktoren beeinflusst, die modell-, datensatz-, instituts- und/oder standardisierungsbedingt sind.
- Wichtig bei Entwicklung und Auswahl von Modellen sind vor allem:
  - Das Bewusstsein des Übertragbarkeitsproblems: Gute Performance in der Entwicklung garantiert keine verlässliche Performance in der Anwendung.
  - Die Nutzung von großen, diversen und unabhängigen Datensätzen für Training und Test, wenn möglich.
  - Das Anwenden und Weiterentwickeln von Methoden zur Verbesserung der Transferierbarkeit, wie z. B. Stain-Normalisierung und NoisyEnsemble.

### Supplementary Information




