# Supplementary material for: The model transferability of AI in digital pathology: Potential and reality
Source: Pathologie (Heidelb). 2024 Feb 19;45(2):124–32. [Article in German] doi: 10.1007/s00292-024-01299-5 (PMC10901943; doi:10.1007/s00292-024-01299-5)
Supplement: Supplementary file 1 [file 292_2024_1299_MOESM1_ESM.pdf]

Onlinezusatzmaterial

Material und Methoden

Datensätze:

Für die Vorhersage von Karzinom- und Normalgewebe Tiles aus Urothelgewebe wurden für das Training diagnostic Slides der TCGA-MIBC (2017) Datenbank gewählt. Dafür wurde ein Subset aus 107 Patienten mit je einem whole slide image (WSI), aus insgesamt 4 Tissue source sites gewählt. Ein exklusiver interner Testdatensatz wurde aus 17 weiteren Patienten der TCGA-MIBC Kohorte gebildet, welche nicht Teil des Trainingssets sind. Die Annotationen wurden mit dem brush-Tool von Qupath erstellt, die Tile Extraction mit der Funktion read_region der python library openslide durchgeführt. Je Klasse (Karzinom- vs. Normalgewebe) wurden hier 40 Tiles a 299x299 Pixel, mit einer Auflösung von 1.0 mpp zufällig aus den annotierten Bereichen „sicher Tumor" und „normal" gezogen. Als externer Testdatensatz nutzten wir 17 Slides von insgesamt 17 Patienten aus einer Kohorte des Dr. Senckenbergischen Insituts für Pathologie (SIP). Hier wurden insgesamt 898 Tiles gezogen. Größe und Auflösung der Tiles stimmen überein. Aufgrund des Annotationstyps (TMA-Markierungen (Sysmex Caseviewer), anstelle von Freihandannotation (Qupath)) weicht hier die Anzahl der möglichen Testtiles pro Patienten vom internen Datensatz ab.

Für die Vorhersage des histologischen small-Duct und large-Duct Typs im intrahepatischen Cholangiokarzinom (iCCA) wurde für das Training ein interner Datensatz des SIP gewählt. Hierbei handelt es sich um einen Datensatz aus 38 small-Duct und 24 large-Duct Patienten. Es wurden pro Patient und Slide je 50 Tiles der Größe 512x512 und einer Auflösung von 1.0 mpp aus dem als „sicher Tumor" annotierten Bereich gezogen. Als externer Testdatensatz dient eine Kohorte der Medizinischen Hochschule Hannover (MHH). Hier werden aus 16 large- und 9 small-Duct Patienten pro Patient 30 Tiles aus dem als „sicher Tumor" annotierten Bereich gezogen.

Farbnormalisierung:

Farbnormalisierung wurde nach Vahadane et al. [27] durchgeführt. Dabei wurde das Referenztile jeweils aus dem Mittleren Stainvektoren nur des Trainingsdatensatzes gewählt, sowohl Training als auch Test wurden daraufhin an das Referenztile angeglichen. Für Abb. 2 wurden Netzwerke ohne Farbnormalisierung trainiert und getestet, alle weiteren Modelle mit Ausnahme der HSV nutzen normalisierte Tiles.

HSV-Augmentierung

Als Alternative zur Farbnormalisierung wurde ebenfalls Hue-Saturation-Value-Augmentation nach Tellez et al. [26] durchgeführt. Hierbei wurden Tiles im Training per Epoche via der skimage Funktion color.rgb2hsv zunächst in den HSV-Raum translatiert und anschließend zu den Werten für hue und saturation ein Zufälliger Wert zwischen -0.1 und 0.1 addiert. Dies entspricht der HSV-light Methode beschrieben durch Tellez et al. Anschließend wurden die

augmentierten Bilder wieder via der skimage Funktion color.hsv2rgb in den RGB-Raum translatiert.

Training der Modelle

Als Framework für die Convolutional Neural Networks (CNN) Modelle wurde Tensorflow + Keras gewählt. Das Basismodell ist ein auf ImageNet vortrainiertes ResNet18, VGG16, Densenet121 und Xception. Verwendet wurde außerdem je ein custom Head welcher aus einem Dense Layer (x256 Nodes, ReLu) einem Dropout (0.5) und Dense Layer (x2 Nodes, softmax). Die Inputsize konnte entsprechend auf 299x299 angepasst werden. Der Optimizer war AdaMax, mit einer Learning Rate von 0.001. Als Loss wurde Binary Crossentropy gewählt.

Für die Vision-Transformer (ViT) wurde die python library ViT-Keras genutzt. Hierbei wurde das Modell vit_b16 verwendet. Genutzt wurden die ImageNet Gewichte sowie ein custom Head welcher aus einem Dense Layer (x256 Nodes, ReLu) einem Dropout (0.5) und Dense Layer (x2 Nodes, softmax) besteht. Die Inpusize des ViT muss ein Vielfaches von 16 sein, daher wurden die Bilder hier auf 288x288 (von 299x299) für Urothelgewebe bzw. 384x384 (von 512x512) bei iCAA geschnitten. Optimizer und Loss entsprechen dem CNN-Training.

Trainiert wurde jeweils 25 Epochen lang, auf dem Urothelkarzinom mit einem binären Tile-Label (Karzinom- und Normalgewebe) beim iCCA auf histologischer Klasse (small-Duct / large-Duct). In jedem Training wurden 10 Patienten des Trainingssets beim Urothelgewebe, sowie 6 bei iCCA (aufgrund geringerer Gesamtpatientenanzahl) als Validierungsset zurückgehalten. Es wurde eine einfache Data-Augmentation via Rotation (90,180,270) bzw. Flip durchgeführt. Ein Callback (tf.keras.callbacks), welcher nur das Modell mit der höchsten Genauigkeit auf dem Validierungsset abspeichert wurde genutzt. Interner und externer Test bleiben dabei durchweg unabhängig vom Training.

Artefakte

Für Abb. 3 wurden Artefakte in den Test und teils in die Trainingsdaten eingebaut. Dafür wurde ein Gauß'scher Noise mit dem Python Modul cv2 und der Funktion cv2.GaussianBlur() auf die Bilder gerechnet. Genutzt wurden dafür Kernel Sizes von 1, 3, 5, 7 und 9. Helligkeit und Kontrast wurden ebenfalls via cv2 mit der Funktion cv2.addWeighted() angepasst. Für den Kontrast wurde der Alpha Wert auf jeweils auf 1.1, 1.3, 1.45, 1.6 und 1.9 (mehr Kontrast). Für Helligkeit wurde der Beta Wert auf jeweils 32, 48, 64, 96 und 127 (heller) gesetzt. Für die JPEG-Kompression wurden die Bilder via der Python-Library Pillow in einem „compressed_stream" (BytesIO) mit entsprechender Kompression gespeichert und anschließend wieder geladen. Als quality-Wert (q-Value) wurden hier je 10, 30, 50, 70 und 90 verwendet.

Ensembles und NoisyEnsembles

Für die Ensembles und NoisyEnsembles in Abb. 4 wurden jeweils 15 Modelle, welche auf leicht variierenden Trainings- und Validierungssets trainiert wurden, zufällig ausgewählt und deren vorhersage durch Abstimmung kombiniert.

Für die NoisyEnsembles wurde im Training beim Urothel-Datensatz jeweils nur eine Klasse pro Patient ausgewählt. Auf dieser wurden 15% Noise eingefügt (heißt das Label wurde umgedreht). Für den iCCA-Datensatz gab es von vornherein nur eine Klasse pro Patient. Dort musste nur der Noise eingefügt werden, in diesem Fall 10%. Anschließend wurde aus den mit Noise trainierten Modellen die NoisyEnsembles gebildet. Dieser Vorgang erfolgte analog für die CNNs sowie für die ViTs.

Abbildungen

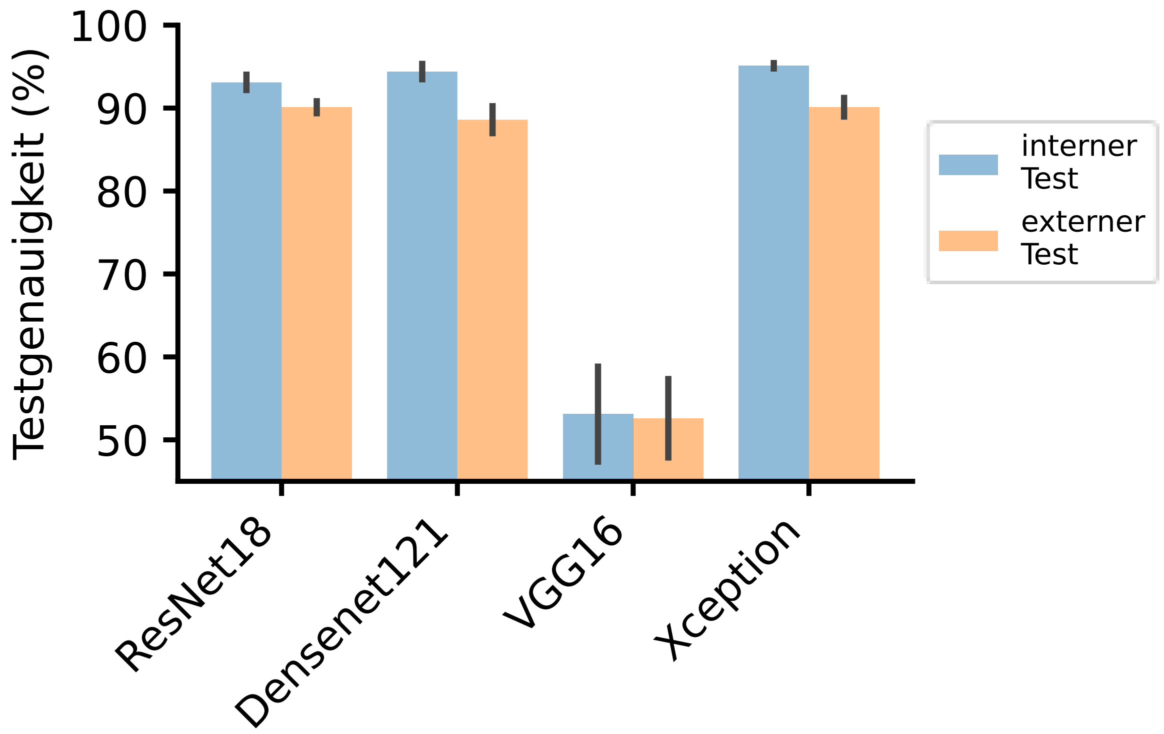

Abb. S1: Performance verschiedener CNN-Architekturen auf Urothelgewebe. Genauigkeiten der Unterscheidung von Karzinom- und Normalgewebe im Urothelkarzinom für je mit ImageNet vortrainierte ResNet18, Densenet121, VGG16 und Xception. Kleinere Modelle wie VGG16 erreichen Genauigkeiten die beinahe dem reinen Zufall entsprechen. Große Modelle erreichen untereinander ähnlich gute Performance, wobei größere Modelle wie Densenet121 gegenüber dem ResNet18 zwar eine höhere interne Testgenauigkeit aber auch eine geringere externe Testgenauigkeit aufweist, was ggf. auf ein leichtes Overfitting hindeuten kann.

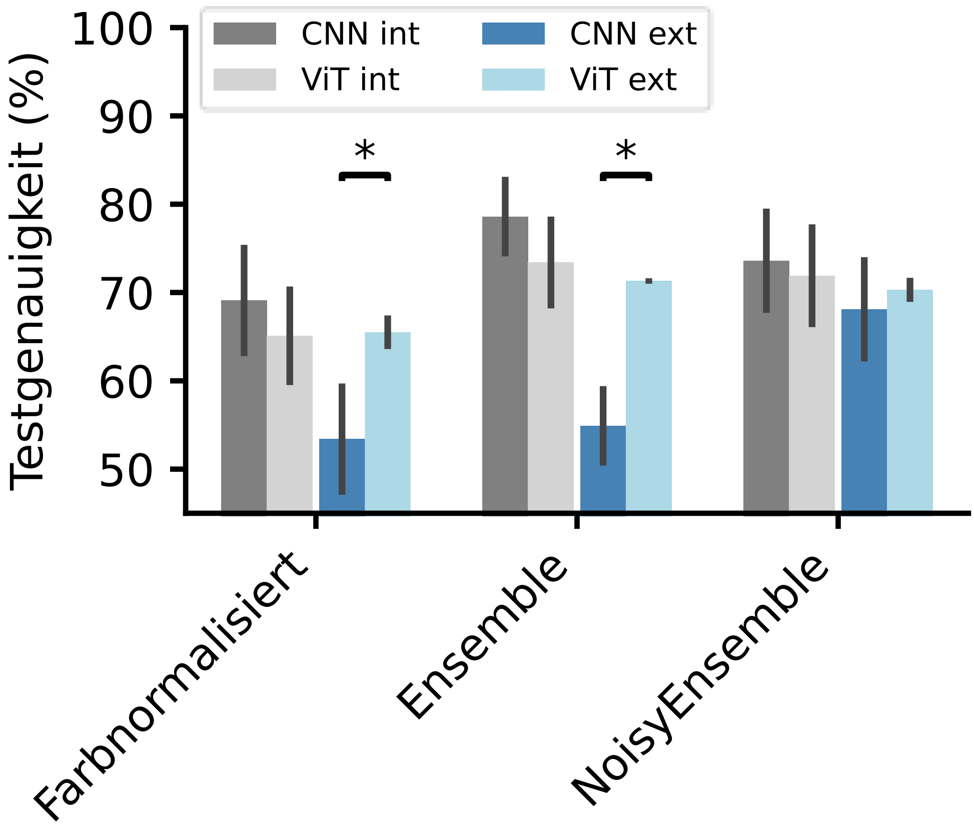

Abb. S2: Vision Transformer haben eine gute Transferierbarkeit im iCCA. Performance der ViT im Vergleich zu CNNs bei der Unterscheidung von histologischen small-Duct und large-Duct Typen im intrahepatischen Cholangiokarzinom im internen (int, SIP) und externen (ext, MHH) Testdatensatz bei den verschiedenen Experimenten: Training von individuellen Modellen farbnormalisierten Bildern, sowie das Training von Ensembles und NoisyEnsembles (jeweils mit Farbnormalisierten Bildern). Hier zeigen bei den ViTs eine höhere Genauigkeit für den externen Test und kleinere Differenzen zwischen internem und externen Test im Vergleich zu den CNNs. Bei internen Tests weisen CNNs höhere Genauigkeit auf. Fehlerbalken zeigen das 95% Konfidenzintervall via Bootstrapping und *zeigen signifikante Unterschiede (nach Mann-Whitney-U-Test; $p<0.05$).
